# Baclofen Potentiates Neurological Impairment in Dialysis Disequilibrium Syndrome

**DOI:** 10.7759/cureus.99018

**Published:** 2025-12-11

**Authors:** Junjing Guo, Latif Salam, Kwesi Blackman, Clinton Brown

**Affiliations:** 1 Medicine, SUNY Downstate Health Science University, New York, USA; 2 Medicine, New York Presbyterian Hospital, New York, USA

**Keywords:** baclofen, dialysis disequilibrium syndrome, encephalopathy, hemodialysis, kidney disease, persistent hiccups

## Abstract

Dialysis disequilibrium syndrome (DDS) is a complication of hemodialysis (HD) that manifests as neurologic symptoms and signs related to osmotic fluid shifts. The drug baclofen is a centrally acting gamma aminobutyric acid (GABA) analog and muscle relaxant that is often used as a treatment for spasticity and as a first-line therapy for hiccups. Baclofen neurotoxicity produces symptoms of generalized CNS depression that can be more prominent in patients with end-stage renal disease (ESRD). We present a case of encephalopathy observed in a 68-year-old male following a first-time dialysis for a patient with ESRD who was initially treated for persistent hiccups with baclofen, in order to examine a possible and previously unreported neurotoxicologic interaction between baclofen and dialysis. Following successful treatment of hiccups due to uremia with baclofen, the drug was discontinued and the patient was administered two sessions of HD for two hours with low blood flow rate. After HD, the patient's blood urea nitrogen (BUN) declined to 50 mg/dL and the patient became profoundly lethargic with accompanying confusion and episodes of agitation, prompting a diagnosis of DDS. After further HD therapy was withheld, the patient’s BUN increased to 87 mg/dL and his mental status returned to baseline within 72 hours. Notably, the reduction in BUN produced by HD was below the suggested threshold level that is likely to cause cerebral edema and also lower than that reported in several case studies of DDS. These findings suggest that in the setting of poor renal excretion in ESRD, a residual level of baclofen remained after HD that was sufficient to lower the magnitude of BUN reduction necessary to elicit the patient’s symptoms of DDS. Therefore, it is recommended that when baclofen and dialysis are co-administered to first-time HD patients, dialysis length and flow rate should not be arbitrarily set or modified but instead titrated to BUN levels so that each daily clearance of BUN is ≤20 mg/dL.

## Introduction

Dialysis disequilibrium syndrome (DDS) is a complication of hemodialysis (HD) that manifests as neurologic symptoms and signs related to osmotic fluid shifts [1]. DDS is attributable to cerebral edema [1] caused by a rapid decline in blood urea nitrogen (BUN), which is abnormally elevated in end-stage renal disease (ESRD) [2]. DDS is a clinical diagnosis, and there is no specific diagnostic test. Patients at the greatest risk for DDS are those with ESRD who are first started on HD or patients who miss consecutive or multiple HD treatments [1]. DDS is rarely reported partly because only its most severe manifestations, such as altered mental status characterized by somnolence, confusion, disorientation, or seizures [3], are recognized as diagnostic criteria [1]. Since milder symptoms of the syndrome, such as nausea, blurred vision, and restlessness [3], are not routinely diagnosed as DDS, it may be more common than is reported [1].

The drug baclofen is a centrally acting gamma aminobutyric acid (GABA) analog and muscle relaxant that is often used to treat spasticity. It can also be considered first line therapy for the treatment of hiccups [4]. Most hiccups are self-limiting, but hiccups lasting 48 hours or more should warrant further investigation. Intractable hiccups is a sign of uremia, which is attributed to the markedly elevated BUN level [5]. Baclofen has well-known neurologic adverse effects, including symptoms of central nervous system (CNS) depression such as drowsiness, muscle weakness, and impaired consciousness, as well as respiratory depression, involuntary muscle jerking movements, and epileptic convulsions [6]. Symptoms of generalized CNS depression caused by baclofen can be more prominent in patients with ESRD [7]. HD has been used to treat baclofen neurotoxicity in patients with impaired renal function [8,9]. In one case, prolonged sinus tachycardia following baclofen was successfully treated with oral propranolol. The tachycardia, which developed during a fall in plasma baclofen levels in the recovery phase, was attributed to a sudden increase in sympathetic activity following a period of depression [6]. Here, we present a case of suspected DDS in a patient with chronic kidney disease (CKD) stage V who received first-time HD after first being treated with a renal-adjusted dose of baclofen for intractable hiccups. The patient became encephalopathic with no neuroradiological signs of brain edema. A possible neurotoxic interaction between baclofen and dialysis is discussed.

## Case presentation

A 68-year-old male with a medical history of hypertension, type II diabetes mellitus, and CKD stage V not on HD presented to the emergency department in November of 2019 with a complaint of persistent hiccups, belching, and burping. Laboratory results showed markedly elevated BUN and serum creatinine. He was admitted to the medicine inpatient service for intractable hiccups and ESRD, which had been diagnosed three months earlier by his nephrologist. The patient reported having intractable hiccups for three days; burping for about one to two weeks; and cough and chills for two days. He denied any subjective fever. On the day of admission, the patient also reported nausea with one episode of clear liquid vomitus. He denied chest pain, shortness of breath, abdominal pain, diarrhea, constipation, dysuria, or hematuria. On presentation, his vital signs included an oral temperature of 36.6°C, heart rate of 99 beats/min, respirations at 18 breaths/min, and blood pressure of 183/79 mmHg. The physical examination was unremarkable. The cardiac exam showed a regular rate and rhythm with no added sounds. Initial laboratory results showed baseline anemia, high-normal potassium, hyponatremia, hypochloremia, hyperglycemia, and markedly elevated BUN and creatinine with a greatly diminished estimated glomerular filtration rate (eGFR) consistent with kidney failure (Table 1). A patient with ESRD who displays impaired consciousness might also be expected to show hyponatremia and anemia, both of which were among the initial findings in this case. However, there were no serum abnormalities in calcium, magnesium, or phosphorus, thereby implicating cerebral edema secondary to DDS, rather than electrolyte disturbances, as a prominent contributing factor to the observed neurological symptoms.

**Table 1:** Initial laboratory results for the hemodialysis patient on admission BUN, blood urea nitrogen; eGFR, estimated glomerular filtration rate

Chemistry Panel	Laboratory Results	Reference	Finding
Hemoglobin	9.2 g/dL	14.0-18.0	Anemia
Potassium	5.1 mmol/L	3.5-5.1	High normal
Sodium	128 mmol/L	136-145	Hyponatremia
Chloride	88 mmol/L	98-107	Hypochloremia
Bicarbonate	22 mmol/L	21-31	Normal
Glucose	182 mg/dL	70-99	Hyperglycemia
BUN	97 mg/dL	70-25	Markedly elevated
Creatinine	11.1 mg/dL	0.70-1.3	Markedly elevated
eGFR	6 mL/min/1.732	≥89	Kidney failure

**Figure 1:**
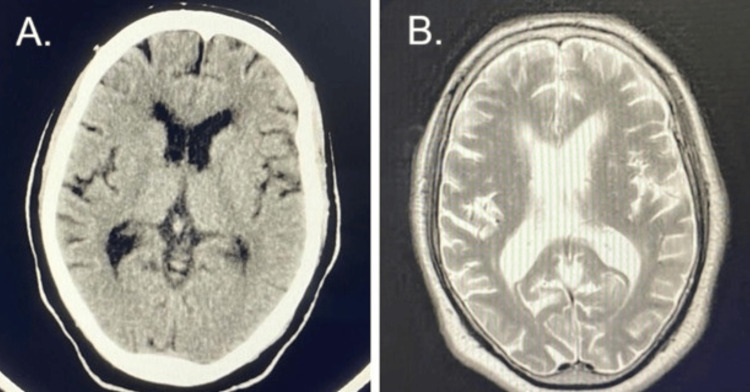
(A) Non-enhanced CT and (B) T2-weighted MRI of the brain in a hemodialysis patient CT, computed tomography; T2WI, MRI, magnetic resonance imaging

For his intractable hiccups likely due to uremia, the patient was initiated on low-dose baclofen. The hiccups resolved, and the baclofen was discontinued within 24 hours. Nephrology was consulted, and on hospital day two, the patient had a short-term HD catheter placed by interventional radiology. On hospital day two, the patient underwent first-time HD for 2 hours with a low blood flow rate (200 mL/min). The most recent laboratory results before dialysis was initiated showed a rise in his BUN to 110 mg/dL and creatinine to 11.3 mg/dL. Following HD, the patient became lethargic with increased nausea and one episode of non-bilious, non-bloody vomiting while BUN had decreased to 71 mg/dL. After a second two-hour HD with low flow rate on the following day, the lethargy became more profound, and the patient became more difficult to arouse. He was also confused and at times agitated. The measured BUN was 50 mg/dL post-dialysis. Since the patient’s BUN level had substantially decreased with each session of HD and he became encephalopathic after initiation of HD, DDS versus baclofen neurotoxicity was suspected. A full septic workup was done and was negative. Neuroimaging studies were performed following the first and second sessions of HD. Computerized tomography and magnetic resonance imaging of the head showed no brain edema or any acute intracranial findings (Figure 1).

His ammonia level was within normal limits at 29 umol/L, and PCO_2_ was within the normal range at 33 mm Hg. The patient was evaluated by the Neurology service, which recommended an electroencephalogram (EEG). The completed EEG showed no seizure activity or any other significant abnormality. The decision was later made with the Nephrology team to withhold further HD therapy, after which the patient’s mental status improved progressively. His serum BUN increased from 50 mg/dL to 56 mg/dL within 24 hours, then to 69 mg/dL within 48 hours, and up to 87 mg/dL within 72 hours. The patient’s mental status returned to baseline, and he had fully recovered within 72 hours. His intractable hiccups did not recur after discontinuing baclofen. After the patient showed full recovery of consciousness 72 hours after cessation of HD, a trial of low-flow-rate HD for two hours triggered another episode of mild encephalopathy. However, the same HD regimen was successfully performed several days later without triggering encephalopathy.

## Discussion

In the presented case, clinical laboratory findings included abnormally high levels of BUN and creatinine, both markers of ESRD. This condition of ESRD most likely predisposed the patient to baclofen-induced neurotoxicity. Once administered, greater than 80% of baclofen is excreted by the kidneys [10] and its normal half-life of 4.5-6.8 h increases in patients with impaired renal function [7]. This explains why patients with CKD or ESRD are more sensitive to baclofen’s side effects than those with normal kidney function.

The patient in the current case received a total of 15 mg baclofen, which falls within the reported dose range for baclofen toxicity among patients with CKD (5-60 mg per day) [11]. He became lethargic with complaints of nausea and vomiting a day later following first time HD. One patient with advanced kidney disease has been reported to develop a state of sleepiness and unconsciousness within 12 hours of administration of a single dose of 25 mg baclofen [8].

Since baclofen is readily removed by HD, we expected that two sessions of HD should have promoted clearance of baclofen to attenuate or eliminate any of its neurotoxic effects, as has been previously reported [8,9]. Therefore, in the current case, the emergence of symptoms of significant neurological impairment following initiation of HD led to a greater suspicion for DDS compared to baclofen neurotoxicity. Patients with DDS report headache, nausea, blurred vision, and restlessness that can progress to somnolence, confusion, disorientation, or mania, and in severe cases seizures, coma, stupor, and death [3]. In the presented case, the patient exhibited nausea, vomiting, and progressive lethargy following dialysis, which is suggestive of DDS. The diagnosis of DDS in this case was made more likely by the finding that BUN levels immediately prior to dialysis (110 mg/dL) were more than fourfold that of the normal range (7-25 mg/dL) and also greater than 100 mg/dL, for which inpatient dialysis is recommended due to increased risk of DDS. It has been suggested that reducing the clearance of BUN for each daily round of HD to effect a more gradual reduction of plasma osmolality can decrease the likelihood of DDS [1]. Based on clinical studies of fluid management for chronic hypernatremia [12,13], it has been recommended that BUN reduction should be targeted so as not to exceed 56-67 mg/dL per day to avoid cerebral edema [1]. A number of studies have shown that when the clearance of BUN over a single dialysis session is substantial, the resulting change in osmolality can precipitate the syndrome. One patient with CKD and no other electrolyte abnormalities was reported to have developed seizures 14 hours after HD when BUN was reduced by 65% (=decrease of 136.5 mg/dL) [14]. However, in the same study, another patient with acute kidney injury treated with HD developed seizures after only a 23% reduction in BUN (=decrease of 49.2 mg/dL) [14]. Another case report described symptoms of DDS occurring with a urea reduction ratio as low as 17% (=decrease of 50.8 mg/dL) [15].

In the presented case, BUN levels were decreased by 35.5% from 110 mg/dL pre-dialysis to 71 mg/dL after the first session of HD and by another 30% from 71 to 50 mg/dL after the second session of HD. The patient exhibited lethargy after the first HD that became even more profound after the second session of HD. Following cessation of HD, the patient’s mental status improved gradually as BUN increased. The largest daily decrease over the two courses of HD was 39 mg/dL, which is below the suggested threshold level that is likely to cause cerebral edema [1] and also lower than that reported in several case studies of DDS [14,15]. Additionally, the patient’s symptoms worsened after the second HD even though BUN level only decreased by 21 mg/dL. One possible explanation of our findings is that a fraction of baclofen remained in the blood after two cycles of two hours of HD. In fact, it has been shown that even in a patient with near end-stage renal disease, 25% of plasma levels remained after five hours of HD. The patient in that case received low dose baclofen just as in the present case, and therapeutic levels of baclofen remained in the circulation after five hours of HD [10]. It has also been reported that patients with decreased renal function have an extended baclofen half-life with more crossing of the blood brain barrier [16]. Therefore, it is quite possible that even after HD, our patient retained significant plasma levels of baclofen that were more able to enter the brain due to ESRD. It is further suggested that this amount of baclofen was sufficient to lower the magnitude of BUN reduction necessary to elicit symptoms of DDS, presumably by causing CNS depression or by exacerbating the neurotoxic effects of dialysis-induced changes in osmolality. Therefore, even after two sessions of HD, our findings suggest that residual levels of the drug may remain in the brain to hypothetically potentiate the neurological symptoms and signs of DDS. In this case report, a modest yet clinically significant intracellular edema undetectable by routine neuroimaging may have led to the observed encephalopathy at a smaller reduction in BUN levels, in combination with the CNS effects of residual baclofen. Following BUN levels carefully after cessation of HD therapy was instrumental in our diagnostic conclusions, which likely would have been strengthened by additional or more sensitive neuroimaging studies over a longer time course. Therefore, it is recommended that when baclofen and dialysis are co-administered to first time HD patients, dialysis length and flow rate should not be arbitrarily set or modified but instead titrated to BUN levels so that each daily clearance of BUN is ≤20 mg/dL. It is also recommended that in the setting of HD in ESRD, caution should be exercised in the use of any other medications that cause generalized CNS depression.

## Conclusions

In summary, we report findings suggesting that following a first-time HD of an ESRD patient with a high BUN level, even a mild form of DDS can be potentiated by prior treatment with baclofen to induce a moderate to severe encephalopathy. Although dialysis can promote the clearance of baclofen and at least partly reverse its neurotoxic effects, residual levels of baclofen may still persist in the brain following HD due to its incomplete removal from the blood by HD and poor renal excretion in the setting of ESRD. Therefore, when baclofen and dialysis are co-administered, it is critically important to not arbitrarily set or modify dialysis length and flow rate but instead carefully titrate these parameters to a daily BUN clearance ≤20 mg/dL to avoid DDS and its possible neurotoxic interaction with baclofen.
